# Simultaneous Medullary and Papillary Thyroid Carcinomas: Personal Experience Report and Literature Review

**DOI:** 10.3390/jcm14041382

**Published:** 2025-02-19

**Authors:** Nadia De Falco, Massimo Agresti, Massimo De Falco, Pasquale Sperlongano, Giancarlo Moccia, Pasquale Luongo, Alessio Cece, Francesco Bove, Francesco Miele, Alfredo Allaria, Francesco Torelli, Paola Bassi, Antonella Sciarra, Stefano Avenia, Paola Della Monica, Federica Colapietra, Marina Di Domenico, Ludovico Docimo, Domenico Parmeggiani

**Affiliations:** 1 Department of Integrated Activities in Surgery, Orthopedy and Hepato-Gastroenterology, Univeristary Policlinico “Luigi Vanvitelli”, 50134 Naples, Italy; nadia.defalco@unicampania.it (N.D.F.); massimo.agresti@unicampania.it (M.A.); massimo.defalco@unicampania.it (M.D.F.); pasquale.sperlongano@unicampania.it (P.S.); giancarlo.moccia@unicampania.it (G.M.); pasquale.luongo@unicampania.it (P.L.); alessio.cece@unicampania.it (A.C.); francesco.bove@unicampania.it (F.B.); francesco.miele@unicampania.it (F.M.); alfredo.allaria@unicampania.it (A.A.); francesco.torelli@unicampania.it (F.T.); paola.bassi@unicampania.it (P.B.); antonella.sciarra@unicampania.it (A.S.); 2 Department of Precision Medicine, University of Campania “Luigi Vanvitelli”, 50134 Naples, Italy; stefano.avenia@unipg.it (S.A.); paola.dellamonica@unicampania.it (P.D.M.); federica.colapietra@unicampania.it (F.C.); marina.didomenico@unicampania.it (M.D.D.); 3 Department of General And Specialistic Surgery, Universitary Policlinico “Luigi Vanvitelli”, 50134 Naples, Italy; ludovico.docimo@unicampania.it

**Keywords:** papillary thyroid carcinoma, medullary thyroid carcinoma, total thyroidectomy, lymphadenectomy

## Abstract

While the frequency of papillary thyroid carcinoma (PTC) has increased in recent decades, both due to improvements in diagnostic procedures and a real, effective percentage increase in cases, the frequency of medullary thyroid carcinoma (MTC), however, has remained almost unchanged, representing 3–5% of thyroid cancer cases. Our experience relates to the observation of cases with the synchronous presence of PTC and MTC, also in chronic autoimmune thyroiditis, and this led us to carry out a brief review of the literature on the subject, with the aim above all of identifying the most correct postoperative therapeutic process.

## 1. Introduction

While the incidence of papillary thyroid carcinoma (PTC) has risen in recent decades, driven both by advancements in diagnostic techniques and a genuine increase in cases, the frequency of medullary thyroid carcinoma (MTC) has remained largely stable, accounting for 3–5% of all thyroid cancer cases [1,2,3]. This trend underscores a notable divergence in the epidemiology of these two thyroid malignancies. The development of high-resolution ultrasound and fine-needle aspiration cytology has certainly contributed to improved detection of PTC. However, the stable incidence of MTC may reflect the consistency of its risk factors, predominantly linked to genetic mutations in the RET (REarranged during Transfection) proto-oncogene and its association with hereditary syndromes, such as multiple endocrine neoplasia type 2.

The occurrence of PTC and MTC in conjunction has been reported with increasing frequency, yet this synchronous association remains exceptionally rare, constituting less than 1% of all thyroid cancer cases [4,5]. This rarity presents challenges not only in diagnosis but also in the establishment of standardized treatment protocols. Despite its infrequent occurrence, the identification of concurrent PTC and MTC has become more achievable, owing to advancements in pathological examination and molecular analysis. This progress has spurred greater interest in determining whether this coexistence results from shared pathogenic mechanisms or merely represents a coincidental occurrence of two distinct malignancies.

Alongside individual case reports [6,7,8], only a small number of multicenter or institutional studies have been published in the past decade [3,9,10], providing insight into the diagnosis and treatment strategies for this rare association. These studies have yielded valuable information, particularly regarding the molecular characteristics of the tumors and their implications for personalized treatment approaches [3,6,7,8,9,10]. Nevertheless, the precise origin of this dual malignancy remains a topic of ongoing debate. One hypothesis suggests a “common” origin from totipotent stem cells capable of differentiating into both follicular and parafollicular cell lineages, while another proposes a “mixed” origin, indicating that the coexistence may be a coincidence rather than the result of a shared oncogenic pathway [3,6,7,8,9,10].

Our experience includes a case of synchronous PTC and MTC occurring within the context of chronic autoimmune thyroiditis, a particularly intriguing association, as autoimmune thyroiditis is more commonly linked to PTC and seldom to MTC. The inflammatory microenvironment associated with chronic thyroiditis may influence tumorigenesis, potentially creating conditions conducive to neoplastic transformation in thyroid tissue. This rare case prompted us to conduct a brief review of the literature on the subject, with a primary aim of identifying the most appropriate postoperative therapeutic approach.

The presence of both tumor types may require a hybrid approach, combining elements of both treatment protocols to optimize outcomes. Future research, particularly large-scale multicenter studies, is crucial to clarify the pathogenesis and optimal management of synchronous PTC and MTC. Molecular profiling and advanced imaging techniques are likely to play key roles in distinguishing between mixed and common origins and in identifying potential therapeutic targets for personalized medicine in these complex cases.

## 2. Our Experience

Over the past 30 years of experience in Oncological Thyroid Surgery—initially at the Division of Thyroid Surgery, later the V Division of Thyroid Surgery, and currently at the Endocrine Surgery Unit of the I Polyclinic of the University of Campania “Luigi Vanvitelli”—we have encountered three cases of synchronous papillary thyroid carcinoma (PTC) and medullary thyroid carcinoma (MTC). These included a 52-year-old male patient and two female patients aged 38 and 64 years, respectively. None had a family history of thyroid disease, and all were non-smokers. They came to our attention during follow-up for chronic autoimmune thyroiditis; in two cases, slight hypothyroidism had been corrected with low-dose L-Thyroxine. All three presented with recent-onset thyroid nodules: in two cases affecting the right lobe and in one case the left lobe. The nodules were hard, mobile upon swallowing, barely palpable, and with no evidence of loco-regional lymphadenopathy.

**Figure 1:**
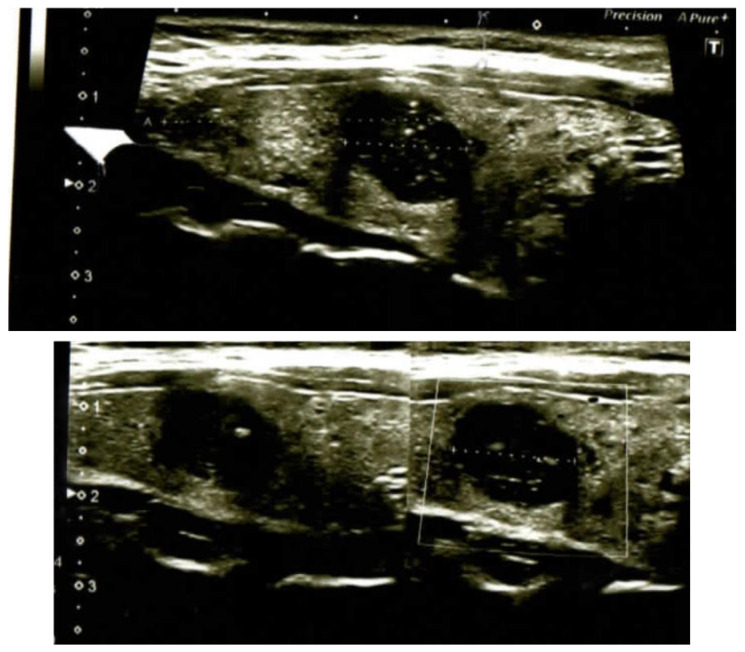
Echo images with simultaneous medullary thyroid carcinoma (in the middle) and papillary thyroid carcinoma (the smaller node).

Ultrasound findings, consistent with autoimmune thyroiditis, revealed hypoechoic nodules with specific characteristics: in case 1 (M, 52 years), a nodule measuring 18 × 9 mm in the upper third of the right lobe and an 8 mm micronodule in the lower third of the same lobe; in case 2 (F, 64 years), a 25 × 16 mm macronodule in the middle third of the left lobe and two micronodules in the right lobe measuring 6 mm and 5 mm, respectively; and in case 3 (F, 38 years), a 13 × 10 mm nodule in the central right lobe and a 6 mm apical micronodule. Predominantly perilesional vascularization (pattern II or IIIa according to Lagalla) was observed in all cases. No pathological cervical lymph node involvement was detected, although cases 2 and 3 presented some hyperplastic nodes with reactive appearance (Figure 1).

Thyroid function tests and thyroglobulin levels were within normal ranges in all cases, while antibody titers (Tg-Ab and TPO-Ab) were elevated. Fine needle aspiration biopsy (FNAB) performed on the dominant nodules revealed hypercellular samples with notable cytoarchitectural variability. Findings included solid nests, branching clusters, microfollicles, dispersed cells of variable size and shape, and scant colloid. These results suggested malignancy, prompting cytological re-evaluation for immunohistochemical analysis in cases 2 and 3 and calcitonin dosage in all cases. Calcitonin levels were markedly elevated (case 1: 158 pg/mL, case 2: 230 pg/mL, case 3: 332 pg/mL; normal value < 6 pg/mL), corroborating the suspicion of medullary carcinoma. CEA levels were at the upper normal limit in all cases.

Molecular analysis of DNA showed no mutations in the analyzed exons, indicating a sporadic form of MTC. However, RNA analysis was inconclusive due to inadequate samples. Following discussions with the patients, it was decided to proceed with total thyroidectomy and central compartment lymphadenectomy (level VI). Postoperative recovery was uneventful, with discharge on the second or third postoperative day, and all patients were started on L-Thyroxine replacement therapy (100 μg/day).

Histological examination confirmed dual malignancy in all cases. Medullary carcinoma was identified in the dominant nodules (case 1: 18 mm right; case 2: 25 mm left; case 3: 13 mm right), with confinement to the thyroid parenchyma and negative surgical margins. Immunohistochemical analysis demonstrated positivity for calcitonin, synaptophysin, chromogranin, and CD56, and negativity for PAX8. The proliferative index (Ki-67) was below 15%, with focal stromal desmoplasia.

The smaller nodules (case 1: 8 mm right; case 2: 6 mm and 5 mm multifocal right; case 3: 6 mm right) exhibited features of classic papillary microcarcinoma, with focal extracapsular extension (0.1–0.2 mm in cases 1 and 3). Coexisting thyroiditis was observed in all cases. Periglandular lymph nodes were free of neoplasia, except for one metastatic lymph node from PTC in case 3 (level VI). Pathological staging was established for each carcinoma type: pT1bN0 for MTC and pT1aN1a for PTC. Given the presence of the papillary component, all patients underwent radioactive iodine therapy (Table 1).

The coexistence of PTC and MTC, while rare, presents a significant diagnostic and management challenge. These malignancies originate from distinct cell types: follicular cells in PTC and parafollicular C cells in MTC. A multidisciplinary approach integrating imaging, cytology, biochemical markers (e.g., calcitonin and CEA), and molecular studies is critical for accurate diagnosis. The absence of DNA mutations suggests sporadic MTC, while inconclusive RNA analysis underscores the need for improved diagnostic techniques. Total thyroidectomy combined with central compartment lymphadenectomy addresses both pathologies effectively. The histological findings confirmed minimal extracapsular extension for PTC and confinement of MTC to the thyroid. Radioactive iodine therapy tailored to PTC provides an additional therapeutic benefit. Long-term follow-up is essential to monitor for recurrence or metastasis, given the distinct biological behaviors of these carcinomas.

Follow-up ranged from 3 to 15 ys. The 3–5–10-year controls showed normal calcitonin (<1.0 pg/mL), Htg < 0.3 ng/mL and negative ultrasound (no residue in the lodge, no pathological lymphadenopathy). We did not see any recurrence of pathology during the follow-up.

## 3. Discussion

Despite the overall increase in the incidence of thyroid carcinomas over recent decades, the co-occurrence of PTC and MTC remains exceedingly rare in clinical practice. Since Lamberg’s initial report in 1981 [12], numerous case studies have been published. However, the majority of these are limited to individual cases or small case series, with only a few multicenter or institutional studies conducted [3,9,10]. Although these studies provide valuable insights, they often present conflicting results when compared to those for PTC or MTC alone, primarily due to the rarity of the condition. Furthermore, the validity of these findings is often compromised by variability in diagnostic and therapeutic approaches, as well as the limited sample sizes from individual institutions [3,6,7,8,9,10]. This ongoing debate revolves around whether the simultaneous occurrence of PTC and MTC is a random event or the result of shared pathogenic mechanisms.

In cases of concurrent PTC and MTC, the two tumor components are frequently spatially distinct, separated by normal thyroid tissue within the same lobe or found in separate lobes, often with well-defined borders [6,7,8]. This observation supports the hypothesis of independent tumorigenesis. Conversely, the “collision theory” suggests that two histologically distinct tumors may invade each other at the same site, forming a single mass within the thyroid gland [10,13]. This theory is further substantiated by the high prevalence of papillary microcarcinomas (PTMCs) in the general population. Interestingly, in cases of PTC and MTC association, the papillary component is often represented by microcarcinomas in approximately 80% of cases [2,14,15]. Alternatively, the presence of “mixed” forms, where a single lesion exhibits both PTC and MTC morphologies and immunoreactivity, suggests a shared origin from totipotent stem cells, supported by evidence of potential common genetic alterations [16,17].

At the genetic level, sporadic MTC is caused by a germline mutation in the RET proto-oncogene in 30–50% of cases, whereas PTC is primarily linked to somatic mutations in the BRAFV600E gene. Only 20–40% of PTCs exhibit abnormalities in RET expression [18,19,20]. Despite ongoing efforts to identify shared oncogenic pathways, genetic analyses have yet to reveal a distinct mutational pattern or common genetic inheritance between the two neoplasms, yielding inconsistent results [9,21]. Nevertheless, the involvement of the RET proto-oncogene, through genetic rearrangements in PTC and point mutations in MTC, remains a plausible connection between the two malignancies [22]. Even rarer is the concurrent presence of both malignancies alongside Hashimoto’s thyroiditis, as observed in our case. This association is thought to result from specific gene rearrangements [23]. It is well established that approximately 25% of PTC cases coexist with Hashimoto’s thyroiditis, a correlation supported by shared biomarkers, genetic mutations, and immune-mediated pathways involving Cd3+, Cd4+, and Th17 cells [24]. However, the mechanisms underlying carcinogenesis in cases of concurrent PTC and MTC remain unclear.

Cytological diagnosis of MTC can be particularly challenging, especially in cases of multinodularity where PTC has already been confirmed. As noted in our experience, fine-needle aspiration biopsy (FNAB) may reveal features suggestive of MTC, such as spindle-shaped cells, anisonucleosis, and dense cytoplasm. When these features are observed, immunohistochemical testing or serum calcitonin measurements are crucial. Calcitonin, which has been shown to be more sensitive and specific than cytology, plays a pivotal role in distinguishing MTC from other thyroid lesions [25]. The rarity of PTC/MTC association, coupled with the lack of homogeneity in data from multicenter studies and the absence of prospective studies, has hindered the development of consensus guidelines regarding surgery, prognostic factors, and management strategies [9,26]. However, a general consensus suggests that patients with concurrent MTC and PTC should undergo radical surgery due to the greater aggressiveness of MTC compared to PTC. Total thyroidectomy with bilateral central lymph node dissection is considered the standard surgical treatment, with additional neck dissection performed based on imaging findings and serum calcitonin levels [27,28,29,30,31].

The literature indicates that most PTCs associated with MTC are low-risk papillary microcarcinomas, often discovered incidentally, whereas the stage of MTC at diagnosis is the primary prognostic factor affecting progression. Early diagnosis of small MTCs provides the best opportunity for radical cure. In contrast, late-stage MTC (where 60% of patients exhibit lymph node involvement if the tumor exceeds 2 cm) and inadequate surgical treatment (e.g., total thyroidectomy without central compartment lymphadenectomy) significantly worsen outcomes [32,33]. In our case, the dominant tumor was a stage PT1bNo MTC, while the papillary component was an incidental PT1aN1a tumor that required adjuvant radioiodine therapy. Serial follow-ups over one year revealed no evidence of disease recurrence in laboratory tests (CT, CEA, Htg) or imaging studies. This case underscores the importance of thorough pathological examination to detect the concurrent presence of PTC and MTC, enabling early diagnosis, particularly of MTC, and improving prognosis. Further genomic studies are needed to identify molecular signatures of concurrent PTC/MTC, potentially paving the way for more personalized treatment approaches.

Preoperative diagnosis of synchronous medullary thyroid carcinoma (MTC) and papillary thyroid carcinoma (PTC) is nearly impossible, and the subsequent therapeutic approach can become challenging. However, recent advancements in high-level paraclinical investigations have proven valuable in raising suspicion for thyroid malignancy and detecting such cases.

In patients presenting with multiple thyroid nodules, comprehensive evaluation is essential for identifying malignancy. Ultrasound remains the cornerstone of thyroid nodule assessment, with suspicious nodules typically exhibiting characteristics such as microcalcifications, irregular margins, hypoechogenicity, and increased vascularity. When multiple nodules exhibit these features, fine-needle aspiration biopsy (FNAB) should be considered for all nodules meeting the criteria, as relying on the largest or most suspicious nodule alone may result in missed diagnoses of synchronous malignancies [34,35]. Subsequent laboratory tests, including serum calcitonin and carcinoembryonic antigen (CEA) levels, are essential for detecting MTC, while thyroglobulin measurements can aid in evaluating PTC. The utility of advanced molecular and genetic analyses, such as microRNA profiling, has gained increasing recognition. Specific microRNAs have been identified as potential biomarkers for distinguishing between benign and malignant thyroid lesions and could offer additional diagnostic value in complex cases [36,37]. Moreover, recent technological advances have introduced novel imaging techniques and computational tools. For instance, multiparametric ultrasound and elastography offer enhanced characterization of thyroid nodules, improving preoperative risk stratification. In combination with traditional methods, these approaches enable a more comprehensive evaluation, reducing the likelihood of overlooking synchronous malignancies [38].

Given the rarity and complexity of synchronous MTC and PTC, preoperative detection remains a challenge. However, integrating high-level paraclinical investigations, systematic biopsy protocols, and molecular testing into clinical practice can significantly enhance diagnostic accuracy. This approach not only aids in identifying malignancies but also facilitates tailored therapeutic strategies, ultimately improving patient outcomes. By addressing these aspects and incorporating the suggested references, this paper aims to provide valuable insights into the preoperative detection of synchronous thyroid malignancies and highlight advancements that may guide improved clinical practice.

## Figures and Tables

**Table 1:** Staging of AJCC/TNM (American Joint Committee on Cancer/Tumor-Nodes-Metastasis) 8th Edition [11].

Stage	Medullary Thyroid Carcinoma	Papillary Thiroid Carcinoma
Case 1	pT1b N0	pT1a N0
Case 2	pT2N0	pT1am N0
Case 3	pT1bN0	pT1a N1a

## Data Availability

The datasets used and/or analyzed during the current study are available from the corresponding author on reasonable request.
